# Enhanced marine burial of terrestrial organic carbon through the Palaeocene–Eocene Thermal Maximum

**DOI:** 10.1038/s41561-026-02012-2

**Published:** 2026-06-16

**Authors:** Gordon N. Inglis, Jordon D. Hemingway, Richard G. Stockey, Emily H. Hollingsworth, Paul J. Valdes, Alex Farnsworth, Felix J. Elling, Marcus P. S. Badger, Xiaoxiao Zhao, Richard D. Pancost, Ann Pearson, Thomas M. Gernon, Robert G. Hilton

**Affiliations:** 1 School of Ocean and Earth Science, University of Southampton, Southampton, UK; 2 Geological Institute, Department of Earth and Planetary Sciences, ETH Zurich, Zurich, Switzerland; 3 School of Geographical Sciences, University of Bristol, Bristol, UK; 4 State Key Laboratory of Tibetan Plateau Earth System, Environment and Resources, Institute of Tibetan Plateau Research, Chinese Academy of Sciences, Beijing, China; 5 Leibniz-Laboratory for Radiometric Dating and Isotope Research, Christian-Albrechts-University of Kiel, Kiel, Germany; 6 School of Environment, Earth and Ecosystem Sciences, The Open University, Milton Keynes, UK; 7 Organic Geochemistry Unit, School of Earth Sciences and School of Chemistry, Cabot Institute for the Environment, University of Bristol, Bristol, UK; 8 Department of Earth and Planetary Sciences, Harvard University, Cambridge, MA USA; 9 GFZ Helmholtz Centre for Geosciences, Potsdam, Germany; 10 Department of Earth Sciences and School of Geography and the Environment, University of Oxford, Oxford, UK

**Keywords:** Palaeoclimate, Carbon cycle

## Abstract

The Palaeocene–Eocene Thermal Maximum (PETM) occurred ~56 million years ago and was characterized by large-scale carbon release and transient warming. Erosion and subsequent burial of terrestrial organic carbon in marine sediments could have sequestered organic carbon during the PETM—stabilizing global carbon and climate systems—yet direct evidence for this process is lacking. Here we present source-specific biomarker records from five globally distributed shallow marine sites and show that vascular plant and soil organic carbon contributed ~40–95% of total organic carbon in coastal sediments during the PETM. This is higher than modern marine sediments (~12–20% of total organic carbon) and indicates greater terrestrial organic carbon delivery to marine environments during warmer climates. We find that terrestrial organic carbon burial fluxes can increase ~10-to-50-fold during the PETM due to enhanced physical erosion and higher coastal sedimentation rates, implying that marine burial of terrestrial organic carbon sequestered excess carbon released during the PETM. Palaeoclimate model simulations do not account for enhanced delivery of terrestrial organic carbon into the marine realm and are thus missing an important carbon sink. Terrestrial organic carbon burial could act as a negative feedback during other hyperthermals and may aid the long-term (>10,000-year) recovery of the Earth system.

## Abstract (web summary)

The burial of terrestrially derived organic carbon in coastal marine sediments greatly increased during the Palaeocene–Eocene Thermal Maximum, probably helping to draw down a large portion of carbon released during the hyperthermal event, according to biomarker records from five sites.

## Main

The geological record is punctuated by transient warming events (hyperthermals) of differing rates and magnitudes^1^. Hyperthermals are characterized by rapid warming with a relatively abrupt onset (1,000 to 10,000 years) followed by an extended duration (>100,000 years) and input of ^13^C-depleted carbon into the ocean–atmosphere system, recorded as a negative carbon isotope excursion (CIE). The Palaeocene–Eocene Thermal Maximum (PETM) is the largest hyperthermal of the Cenozoic Era and is characterized by an approximate doubling of atmospheric CO_2_ (from ~1,200 to ~2,500 ppm)^2^, a negative CIE (about 4 ‰)^3^ and transient global warming (4–6 °C rise in global temperatures over 10,000 years)^4^. The PETM has provided unique insights into the potential drivers of CO_2_ release^5^ and how the Earth system responds to geologically rapid warming events^1^. Although the magnitude of cooling during the recovery of the PETM is well constrained^2,3,5^, the processes governing the recovery of the climate system during the PETM and other hyperthermals remain unclear.

Over geological timescales, carbon dioxide (CO_2_) can be removed from the ocean–atmosphere system via silicate mineral weathering and/or organic carbon (OC) burial^6^. Silicate weathering coupled to carbonate burial is an important atmospheric CO_2_ sink over relatively long (>10^5^ to 10^6^ year) timescales^6^. This mechanism is thought to respond and contribute to major climate transitions during the Phanerozoic^7^, acting as a stabilizing negative feedback to global warming^8^. Silicate weathering is also important over shorter PETM-relevant timescales (~10^4^ years) with a prediction that weathering fluxes increased by ~35–60 % during the PETM relative to pre-event values^9,10^. This increase has been interpreted to reflect more intense mineral weathering and a shift towards higher physical erosion rates due to changes in hydroclimate^9^, both of which are consistent with increased nutrient and clay delivery into coastal environments^11^. Increasing nutrient delivery can stimulate marine primary productivity, which could lead to enhanced burial of marine OC during the PETM^12^. However, marine burial of terrestrial OC has received little attention, despite currently removing as much carbon per year (an estimated 41–75 megatons of carbon per year; MtC yr^−1^)^13^ as silicate weathering coupled to carbonate precipitation (an estimated 47−72 MtC yr^−1^)^14^.

Modern river data^15^ and lake sediment records^16^ suggest that the erosional flux of terrestrial OC is moderated by hydroclimate. This implies that marine burial of terrestrial OC is a negative carbon cycle feedback that could operate during the PETM alongside silicate weathering^9^ and marine OC burial^12^. However, marine burial of terrestrial OC has been interpreted to either increase^17^ or decrease^18^ during the PETM. This contradictory evidence suggests limitations with the current methodologies that we use to fingerprint terrestrial OC in marine sediments. Two-endmember mixing models are frequently used to reconstruct the proportion of terrestrial versus marine organic carbon in marine sediments (for example, ref. ^18^); such models assume that all terrestrial OC is a single homogeneous pool. However, terrestrial OC is demonstrably a heterogenous mixture of vascular plant and soil OC^19^, and mixing models that fail to distinguish between these two pools underestimate modern terrestrial OC burial in marine sediments by 40–85% (ref. ^20^).

In this study, we employ a novel data-modelling approach to resolve these challenges and evaluate the role of the terrestrial OC cycle as a negative climate feedback during transient warming events. We trace the source-to-sink delivery of vascular plant and soil OC from land to sea by compiling and generating source-specific biomarker records from thermally immature^21,22^ shallow marine PETM sites in the New Jersey Margin, Tanzania, Denmark, the Tasman Sea and the High Arctic. We use a three-endmember mixing model to apportion the sedimentary OC pool into soil, vascular plant and marine OC, overcoming the challenges with previous estimates. These data are combined with measurements of total OC, dry bulk density and sedimentation rates to quantify vascular plant and soil OC burial fluxes in marine sediments during the PETM. We use fully coupled palaeoclimate models to assess the mechanisms responsible for changes in terrestrial OC export and re-assess the role of terrestrial OC burial in driving the long-term recovery of the Earth system following transient warming.

## Coastal sediments are dominated by terrestrial organic carbon

The relative abundance of lipid biomarkers reveals distinct spatial and temporal patterns in OC provenance before, during and after the PETM. We use the terrestrial–aquatic ratio (TAR) to evaluate the relative abundance of short-vs-long-chain *n*-alkanes^23^. Short-chain *n*-alkanes (C_15_–C_19_) are largely derived from algae/zooplankton (that is, marine OC) whereas long-chain *n*-alkanes (C_27_–C_35_) are derived from vascular plants—the latter can be delivered into the marine realm via atmospheric transport (that is, leaf abrasion) and/or via the erosion of catchments soils^23^. Values near 1 are associated with delivery of vascular plants into the marine realm, whereas values approaching 0 reflect greater input from algae or zooplankton. All sites in our compilation are characterized by TAR values > 0.7^24^. The carbon preference index (CPI) is consistently >1 and typically between ~2–6 (refs. ^21,25^), confirming that thermally immature, vascular plant OC is an important contributor to the marine sedimentary OC pool during the latest Palaeocene, PETM and earliest Eocene.

We additionally use the branched-to-isoprenoid tetraether (BIT) index^22^ to evaluate the relative abundance of branched glycerol dialkyl glycerol tetraethers (brGDGTs) vs Crenarchaeol. Branched GDGTs are typically derived from soil bacteria whereas Crenarchaeol is exclusively derived from largely marine-dwelling, ammonia-oxidizing *Nitrososphaerota*. BIT values approaching 1 are associated with the delivery of soil OC into the marine realm, whereas lower values reflect greater OC input from marine organisms. Before the PETM, BIT indices span a wide range and indicate: (1) a mixture of soil and marine OC burial in the High Arctic (BIT average: 0.42) and Denmark (BIT average: 0.56) and (2) predominantly marine OC burial in the New Jersey Margin (average: 0.19) and Tasman Sea (average: 0.14). During the PETM, BIT indices decrease (Denmark, High Arctic, New Jersey Margin)^3,21,26^ or remain stable (Tasman Sea)^27^. BIT values are only available for Tanzania during the PETM but suggest a gradual increase in soil OC burial during the onset of the PETM^28^.

In addition to soil inputs, brGDGTs may be variably produced in riverine, lacustrine and marine settings. Where available, average #rings_tetra_ values (Methods) are <0.7, confirming that brGDGTs are predominantly sourced from the terrestrial realm. However, as brGDGTs can degrade more quickly than other soil OC tracers (for example, long-chain *n*-alkanes, lignin phenols)^29^, all BIT values should be regarded as conservative minimum estimates for soil-derived OC.

**Fig. 1:**
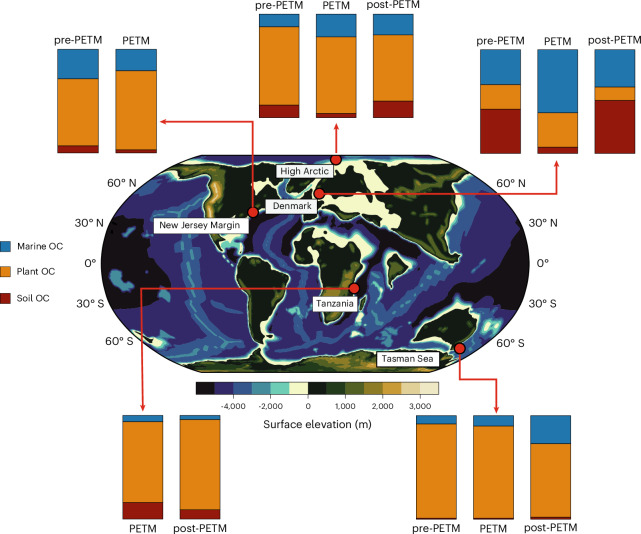
Organic carbon distribution in coastal marine sediments during the PETM. Relative proportion of vascular plant, soil and marine OC in coastal marine sediments during the latest Palaeocene, PETM (onset, body and recovery combined) and/or early Eocene. The locations of each site are plotted on an early Eocene (55 million years ago; Ma) palaeographic map. Basemap adapted from ref. ^52^ under a Creative Commons license CC BY 4.0. Source data

**Fig. 2:**
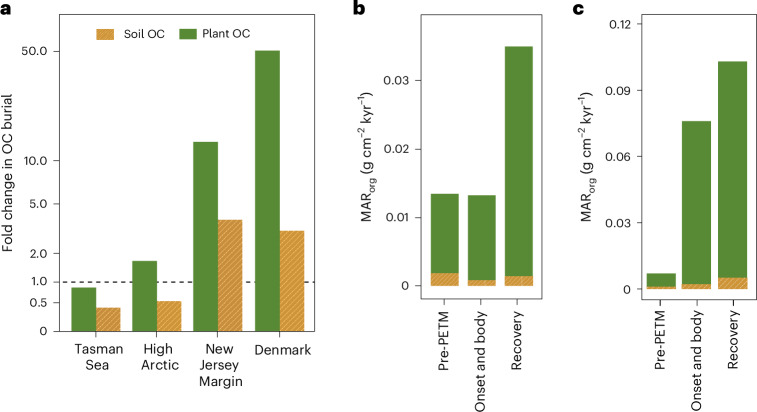
Vascular plant and soil OC burial fluxes in coastal marine sediments during the PETM. **a**, Fold change in vascular plant OC (green solid bar) and soil OC burial (orange striped bar) in marine sediments during the PETM inferred via source-specific biomarker records. *Y* axis is a pseudo-log scale. Values > 1 indicate higher OC burial relative to pre-PETM. Values < 1 indicate lower OC burial relative to pre-PETM. **b**,**c**, Vascular plant and soil OC mass accumulation rates (MARs) increase in the High Arctic (**b**) and New Jersey Margin (**c**) during the PETM recovery. Source data

**Extended Data Fig. 1:**
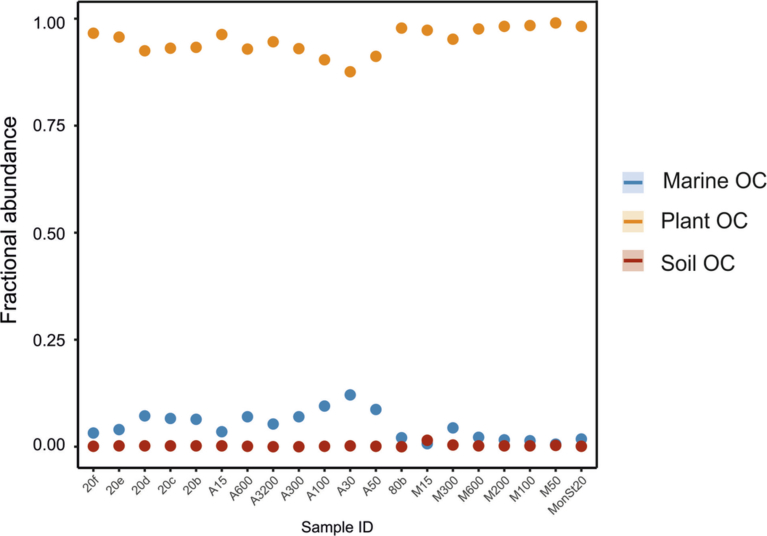
Organic carbon distribution in core-top marine sediments from the Mississippi–Atchafalaya River system. Fractional abundance of plant (orange), soil (red), and marine OC (blue) in core-top sediments from the Mississippi–Atchafalaya River system (Reference #70).

**Extended Data Fig. 2:**
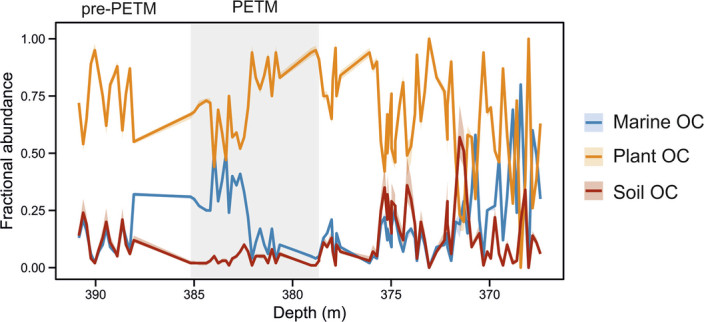
Organic carbon distribution in High Arctic marine sediments during the PETM. Fractional abundance of plant (orange), soil (red), and marine OC (blue) in the High Arctic during the Palaeocene-Eocene. Error bars represent the 25% and 75% quantiles. Grey bar denotes the PETM onset, body and recovery interval.

**Extended Data Fig. 3:**
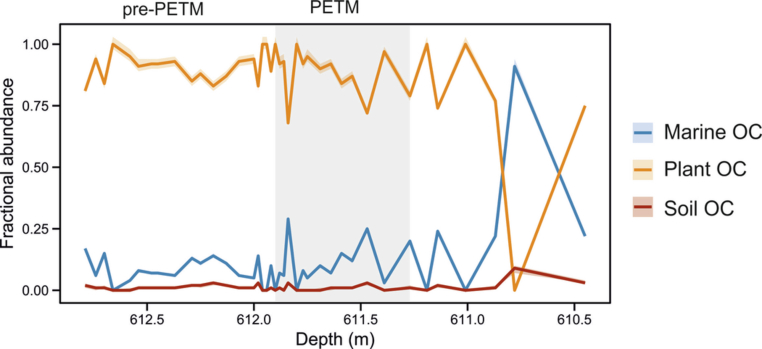
Organic carbon distribution in Tasman Sea marine sediments during the PETM. Fractional abundance of plant (orange), soil (red), and marine OC (blue) in the Tasman Sea during the Palaeocene-Eocene. Error bars represent the 25% and 75% quantiles. Grey bar denotes the PETM onset, body and recovery interval.

**Extended Data Fig. 4:**
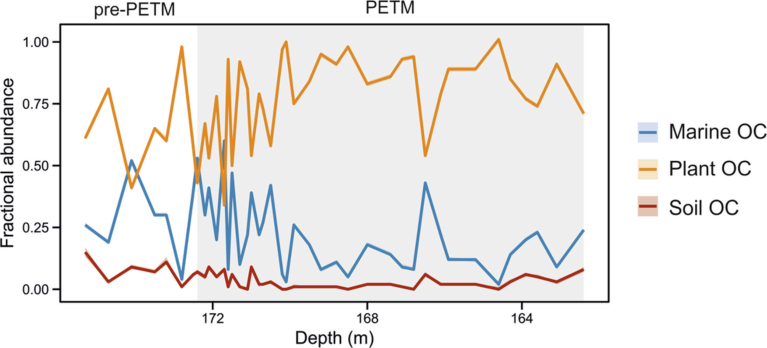
Organic carbon distribution in New Jersey Margin marine sediments during the PETM. Fractional abundance of plant (orange), soil (red), and marine OC (blue) in the New Jersey Margin during the Palaeocene-Eocene. Error bars represent the 25% and 75% quantiles. Grey bar denotes the PETM onset, body and recovery interval.

**Extended Data Fig. 5:**
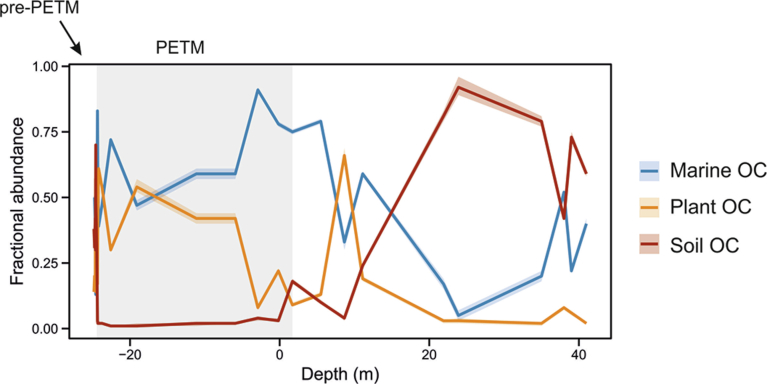
Organic carbon distribution in Denmark marine sediments during the PETM. Fractional abundance of plant (orange), soil (red), and marine OC (blue) in Denmark during the Palaeocene-Eocene. Error bars represent the 25% and 75% quantiles. Grey bar denotes the PETM onset, body and recovery interval.

**Extended Data Fig. 6:**
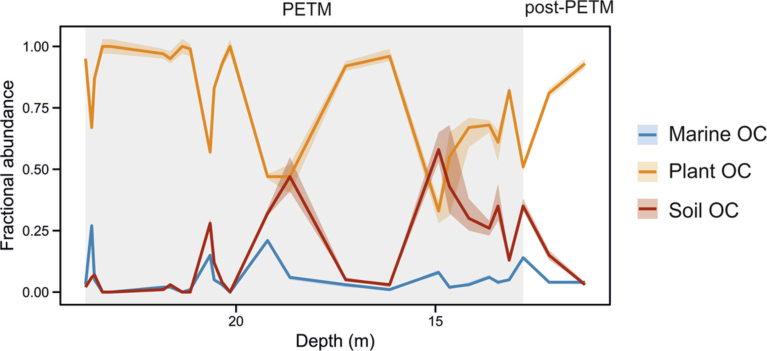
Organic carbon distribution in Tanzania marine sediments during the PETM. Fractional abundance of plant (orange), soil (red), and marine OC (blue) in Tanzania during the Palaeocene-Eocene. Error bars represent the 25% and 75% quantiles. Grey bar denotes the PETM onset, body and recovery interval.

Using a three-endmember mixing model (Methods*;* ref. ^30^) validated in modern coastal sediments (Extended Data Fig. 1 and Methods), we apportion the sedimentary OC pool into soil, vascular plant and marine OC^24^. We find that coastal marine sediments are dominated by vascular plant OC during the latest Palaeocene, PETM and early Eocene (up to 90% of the total (that is, vascular plant + soil + marine) OC pool) with a limited contribution from soil (typically ~5 to 15%) and marine OC (typically ~5 to 20%; Fig. 1 and Extended Data Figs. 2–6). As long-chain *n*-alkanes exhibit strong absorption to clay minerals due to their relatively hydrophobic nature^31^, this may partially explain the dominance of plant > soil OC in each location. The relative proportion of soil vs higher plant vs marine OC in shallow marine sediments does not change dramatically between the latest Palaeocene and PETM (Fig. 2 and Extended Data Figs. 2–6), except for Denmark, where the relative proportion of marine OC increases from ~34 to 62% (Extended Data Fig. 5). The higher proportion of marine OC in Denmark is attributed to localized volcanic ash deposition following eruptions within the North Atlantic Igneous Province and a resulting increase in marine primary productivity^32^.

After accounting for differences in OC content, dry bulk density and sedimentation rates (Methods), we show that between the latest Palaeocene and PETM, vascular plant OC burial flux in marine sediments increased in the High Arctic (1.7-fold change; Fig. 2a) and the New Jersey Margin (13.3-fold change; Fig. 2a) and decreased slightly in the Tasman Sea (0.9-fold change; Fig. 2a). Soil OC burial flux in marine sediments increased in the New Jersey Margin (3.8-fold change; Fig. 2a) and decreased in the Tasman Sea (0.4-fold change; Fig. 2a) and the High Arctic (0.5-fold change; Fig. 2a). Sedimentation rates in Denmark during the latest Palaeocene are uncertain^32^, but there is a dramatic increase in vascular plant OC burial flux (~50-fold change) and soil OC burial flux (~threefold change) between the onset and body of the PETM. As pre-PETM sedimentation rates are unlikely to be higher than those of the PETM onset^28^, we assume that this increase is representative of the PETM (Fig. 2a).

Two sites (New Jersey Margin, High Arctic) have sufficient proxy data with suitable age control to evaluate how vascular plant and soil OC burial flux evolved during the ‘core’ and ‘recovery’ of the PETM (defined following ref. ^25^). In the High Arctic (Fig. 2b) and New Jersey Margin (Fig. 2c), higher plant and soil OC burial flux combined increased 2.7-fold and 1.3-fold between the ‘body’ and ‘recovery’ phase of the PETM, respectively.

Coastal marine sediments can contain other sources of OC not represented in our mixing model (that is, rock OC) and could bias biospheric OC mass accumulation rates (MARs). At four of our five sites, there is an increase in rock OC burial flux during the PETM^25^. However, this is driven by higher sedimentation rates rather than a change in the relative proportion of rock OC vs biospheric OC inferred via hopane thermal maturity ratios^25^. The only exception is Tanzania, which exhibits an increase in the proportion of rock OC^25^. Thus, although our estimated vascular plant and soil OC MARs may represent an upper estimate, the relative changes in MARs between the pre-PETM and PETM are robust.

Overall, our results show that latest Palaeocene and PETM-aged coastal marine sediments are dominated by vascular plant and soil OC, which contribute about 40 to >95% of the total OC pool (Fig. 1). This is higher than observed in modern continental margin sediments (12–20%)^33^ but similar to modern deltaic sediments (43–91%)^33^ with high terrestrial OC burial efficiency (typically >25% but up to 80% in Southeast Asia and the High Arctic^34,35^).

## Hydroclimate modulates terrestrial organic carbon export

Hydroclimate can impact terrestrial OC export in two ways. First, it can control net primary productivity and soil carbon cycle processes, setting the available vascular plant and soil OC in the landscape available for erosion^36^. Second, it impacts hydrological-driven erosion processes, which include a range of mass-wasting and runoff-driven processes that can mobilize terrestrial OC and transport it in river systems^15^. These events can also mobilize large amounts of reactive mineral particles (for example, clay minerals), helping to enhance OC burial further^37^.

To assess how hydroclimate regulates the erosional export and subsequent burial of terrestrial OC in marine sediments, we use the Deep-Time Model Intercomparison Project (DeepMIP) suite of early Eocene model simulations^38^ to simulate the impact of higher CO_2_ on mean annual precipitation (MAP) at each site (Methods). MAP estimates are reported using the multi-model mean (MMM) to minimize the influence of individual model structural biases and highlight the common forced signal (Methods).

**Fig. 3:**
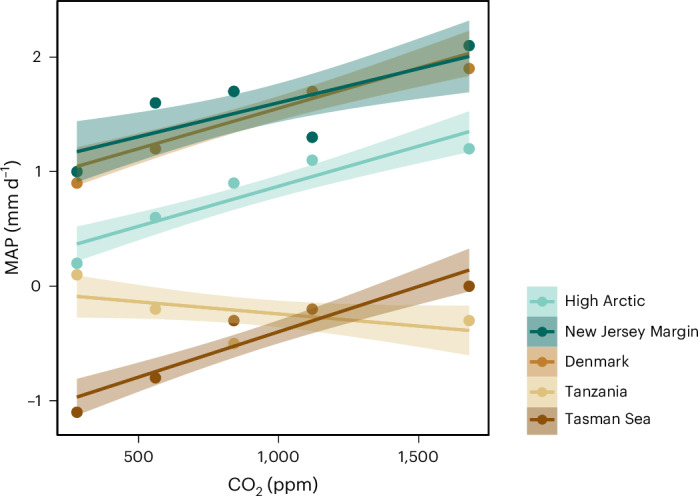
Relationship between atmospheric CO_2_ and MAP in early Eocene climate model simulations for various localities. Each data point represents the multi-model mean from the DeepMIP model ensemble^38^ relative to a pre-industrial ×1 CO_2_ simulation. Colours refer to individual sites in our proxy compilation. Each site is fitted with a linear regression with 68% confidence intervals shown. Source data

The impact of higher CO_2_ on MAP is regionally variable and either increases (Tasman Sea, High Arctic, New Jersey Margin, Denmark; Fig. 3) or decreases (Tanzania; Fig. 3). Tanzania is the only site that exhibits lower MAP estimates under elevated CO_2_, suggesting there are processes beyond MAP that influence the marine burial of terrestrial OC. In modern settings, extreme rainfall events can mobilize exceptionally large volumes of sediment and terrestrial OC^39^. During the PETM, extreme rainfall events in Tanzania intensified by up to ~70% (ref. ^40^), consistent with dramatic fluctuations in biomarkers and mineralogy during the PETM^28^. This mechanism probably explains the strong terrigenous OC signal in Tanzania despite reduced MAP at higher CO_2._

**Fig. 4:**
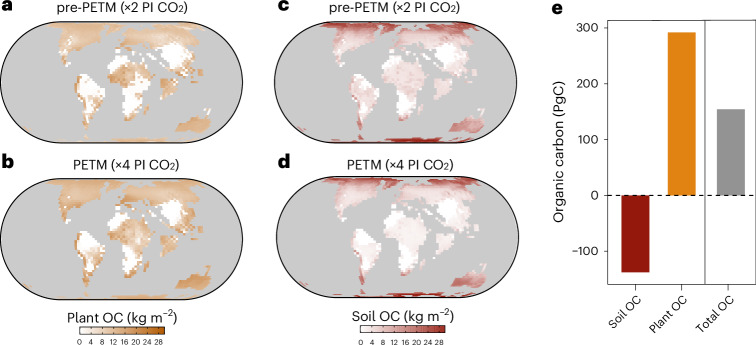
Soil and plant OC stocks on land during the Palaeocene–Eocene inferred via an atmosphere–ocean–vegetation model. **a**–**d**, Plant OC (**a**,**b**) and soil OC (**c**,**d**) stocks during the latest Palaeocene (×2 PI CO_2_) and PETM (×4 PI CO_2_) inferred via HadCM3BL-M2.1 Da. **e**, Difference in soil (red bar), plant (orange bar) and total terrestrial OC stocks (grey bar) between the latest Palaeocene (×2 PI CO_2_) and PETM (×4 PI CO_2_) simulations. Source data

Increasing temperatures and an enhanced hydrological cycle will affect higher plant and soil OC stocks and, potentially, the relative proportion of soil OC versus higher plant OC transported from land to sea. The DeepMIP ensemble prescribes vegetation and soil cover and cannot resolve changes in plant and soil OC stocks during the PETM. To resolve this, we use a fully coupled atmosphere–ocean–vegetation model (HadCM3BL-M2.1 Da (ref. ^41^); Fig. 4) with dynamic vegetation (TRIFFID; Methods) and a terrestrial carbon cycle scheme (MOSES2.1; Methods).

Using this approach, we show that the terrestrial biosphere (that is, vascular plant and soil OC stocks combined; ~2,220 PgC) increases by ~6% (150 PgC) between the latest Palaeocene (×2 pre-industrial (PI) CO_2_) and PETM (×4 PI CO_2_; Fig. 4e). This increase is notable but is modest compared to the mass of carbon thought to be released during the PETM ( ~ 3,000 to >10,000 PgC)^42^. Our estimates are also much lower than the previously inferred increase in the terrestrial biosphere during the PETM inferred using stable carbon isotopes (2,000 PgC)^43^, suggesting that the terrestrial biosphere only acts as a transient sink for excess carbon release. It also implies that changes in the size of OC stocks in the terrestrial biosphere cannot be the only driver of change.

Latest Palaeocene and PETM-aged marine sediments show a high proportion of vascular plant OC compared to soil OC (Fig. 1), which contrasts with many modern-day river systems with high OC export that are instead dominated by soil-derived OC^19^. This may suggest a smaller soil OC pool in past warm climates, consistent with our simulated decline in global soil OC stocks under elevated CO_2_ (Fig. 4c,d) and empirical evidence for enhanced soil OC oxidation in mid-latitude interiors during the PETM^44^.

## Erosion rates are a key driver of terrestrial carbon export

Terrestrial OC burial rates are shown to increase by up to ~10-to-50-fold during the PETM (Fig. 2a) and thus cannot be explained solely by higher terrestrial primary productivity and a larger terrestrial biosphere, as this change is relatively small (+6% relative to latest Palaeocene; Fig. 4e). This is consistent with a comprehensive database of over 40 modern river catchments showing that terrestrial export of soil and vascular plant-derived OC is largely insensitive to the magnitude of terrestrial net primary production^45^. Instead, the primary driver of terrestrial OC export in contemporary environments is the capacity to supply and transport it in river sediment loads (that is, physical erosion rates)^15,45^.

The scaling of terrestrial OC export with erosion is important because it sets sedimentary accumulation rates and OC burial efficiency^13^. This is apparent in modern large, erosive river catchments (for example, Ganges–Brahmaputra rivers fed by the Himalaya erosional system) where high rates of sediment delivery drives rapid accumulation offshore (burial efficiency in the Bay of Bengal approaches ~100%)^46^. It is also true of small mountainous rivers (for example, Taiwan), where short transport lengths and very high turbidity drive rapid sediment delivery to deeper water, and burial efficiencies are >70% (ref. ^47^). As the PETM is characterized by high erosional fluxes^28^, high sedimentation rates^28^ and low oxygen availability^12,28^, these factors together probably explain why PETM sediments are characterized by locally high plant and soil OC burial fluxes (Fig. 2).

## Organic carbon burial in past warm climates is underestimated

Erosion and subsequent burial of terrestrial OC in marine sediments could play a key role in modulating PETM climate. However, widely used biogeochemical models^48^ do not account for input and burial of terrestrial OC in marine sediments. As such, the global significance of our findings remains uncertain. Previous studies using OC mass accumulation rates^12^ suggest that between 40 to 86 MtC yr^−1^ of OC is sequestered in marine sediments during the PETM (assuming a duration of 270 kyr)^12^ (Supplementary Information). This occurs largely in coastal environments and restricted seaways^12^ where the delivery of terrestrial OC is also likely to be high. If we assume that the relative proportion of terrestrial OC in PETM-aged marine sediments ranges between 40 to 95% (Fig. 1), this implies that up to 81 MtC yr^−1^ of OC in PETM-aged sediments is sourced from the terrestrial biosphere. This is comparable to the modern-day burial flux in marine sediments (41–75 MtC yr^−1^)^10^ and demonstrates the powerful role that the terrestrial carbon cycle could play during the PETM, including during the recovery phase (Fig. 2b,c). The burial of terrestrial OC would be consistent with rapid recovery from the initial carbon isotope excursion during the PETM, with terrestrial OC having a more ^13^C-depleted isotope composition than marine OC^13^. Although this could be achieved via regrowth of the terrestrial biosphere^43^, sustained recovery on ${10}^{4}$–${10}^{5}$ yr timescales probably requires transfer and burial of ^13^C-depleted terrestrial OC into sedimentary rocks.

This work offers tantalizing implications for other periods of rapid climate change in the Earth system. Of particular relevance are the early Eocene hyperthermals (for example, ETM2, H2)^2^. These share similar characteristics to the PETM, with evidence for global warming and enhanced physical erosion rates^49^. As these hyperthermals are relatively brief (typical duration ~100 kyr), they require the existence of a negative feedback mechanism that can operate over geologically short (<10^4^ year) timescales. We suggest that terrestrial OC burial in marine sediments could explain the decline in global temperature after successive Eocene hyperthermals. It is also likely that terrestrial OC burial in marine sediments increased during other hyperthermals characterized by large changes in physical erosion rates, including the Permo-Triassic boundary (251.9 million years ago; Ma)^50^, thus implying this feedback may be important throughout the Phanerozoic. Anthropogenic perturbations have also increased the flux of terrestrial OC carbon from land to sea^51^, implying that marine burial of terrestrial OC could potentially aid the long-term (10^3^ to 10^4^ year) recovery of the Earth system after anthropogenic CO_2_ emissions have ceased^1^.

## Methods

### Biomarker analysis and data compilation

Branched GDGT data were compiled from the following PETM-aged sites: (1) Tanzania (TDP Site 14)^28^, (2) New Jersey Margin (Ancora, Site 174AX)^3^, (3) Denmark (Fur)^21^, (4) Tasman Sea (ODP Site 1172)^27^ and (5) the High Arctic (ACEX, IODP Site 302)^26^. Short- and long-chain *n*-alkane data were compiled from (1) Tanzania (TDP Site 14)^28^ and (2) Denmark (Fur)^21^. To complement our data compilation, *n*-alkane and/or branched GDGT distributions were determined for PETM-aged sites in the: (1) High Arctic (*n* = 94; *n*-alkanes only), (2) Tasman Sea (*n* = 41; *n-*alkanes only), (3) New Jersey Margin (*n* = 42; *n-*alkanes and brGDGTs) and (4) Denmark (*n* = 42; brGDGTs only).

Sediments from the High Arctic, Tasman Sea and the New Jersey Margin were freeze dried, homogenized and extracted using a MARS5 microwave-assisted extraction system (ref. ^5^) using (1) dichloromethane:methanol (DCM:MeOH) (1:1, *v*:*v*); (2) DCM:MeOH (9:1, *v*:*v*) and (3) DCM. Solvents were heated for 30 minutes to 100 °C, followed by a hold time of 20 minutes. The extracts were subsequently combined into a total lipid extract (TLE) and separated into five fractions (ref. ^3^ provides more details). Hydrocarbon fractions (containing *n*-alkanes) were analysed using a ThermoFisher Trace 1310 Gas Chromatograph (GC) coupled to a Thermo TSQ8000 Triple Quadrupole mass spectrometer (MS) (GC-MS/MS) at the University of Southampton. Separation was achieved with DB-5 column (30 m × 0.25 mm internal diameter., 0.25 μm film thickness). The GC-MS/MS temperature programme started at 70 °C for 1 minute, increased to 130 °C at 20 °C min^−1^, followed by 300 °C at 4 °C min^−1^, which was then held for 20 minutes. MS scanning occurred between mass-to-charge ratio (*m*/*z*) 50 to 650 Daltons and an ionization energy of 70 eV. Polar fractions (containing branched GDGTs) were analysed using the instruments and conditions described in refs. ^3,53^.

Sediment samples from the Fur Formation were freeze dried and ground using an agate mortar and pestle or an agate ball mill and extracted using a Thermo ASE350 accelerated solvent extraction system at 100 °C and 100 bar in three steps: (1) DCM:MeOH 1:1 (*v*/*v*); (2) DCM:MeOH 9:1 (*v*/*v*); (3) 100% DCM. Each extraction step included (1) preheating for 2 min, (2) holding at 100 °C for 5 min, (3) cool down, before the extract was collected. The three extracts were then combined into a TLE, concentrated under a flow of N_2_ and stored at −20 °C. Branched GDGTs were analysed using a coupled high-performance liquid chromatography (HPLC)-mass spectrometry system (MS) consisting of an Agilent 1260 Infinity II series HPLC and an Agilent 6130 mass spectrometer equipped with an atmospheric pressure chemical ionization interface operated in positive mode. The HPLC method followed ref. ^54^. The MS was operated in selected ion-monitoring mode.

### Biomarker proxies and endmember definitions

The branched-to-isoprenoid tetraether (BIT)^22^ is defined as follows:

1 $$\begin{array}{l}\mathrm{BIT}=(\mathrm{brGDGT}-\mathrm{Ia}+\mathrm{brGDGT}-\mathrm{IIa}+\mathrm{brGDGT}-\mathrm{IIa}{\rm{\mbox{'}}}+\mathrm{brGDGT}-\mathrm{IIIa}\\ +\mathrm{brGDGT}-\mathrm{IIIa}{\rm{\mbox{'}}})/(\mathrm{brGDGT}-\mathrm{Ia}+\mathrm{brGDGT}-\mathrm{IIa}\\ +\mathrm{brGDGT}-\mathrm{IIa}{\rm{\mbox{'}}}+\mathrm{brGDGT}-\mathrm{IIIa}+\mathrm{brGDGT}-\mathrm{IIIa}{\rm{\mbox{'}}}+\mathrm{Crenarchaeol})\end{array}$$

The soil endmember for the BIT index was determined by compiling values in modern peat and soil environments (0.60–1.00; 95% confidence intervals; *n* = 741)^55–65^. The marine endmember was defined using modern distal marine sediments (0.01–0.07; 95% confidence intervals; *n* = 38)^59^.

The terrestrial-to-aquatic ratio (TAR) is defined as follows:

2 $${\rm{TAR}}=({{\rm{C}}}_{29}+{{\rm{C}}}_{31}+{{\rm{C}}}_{33})/({{\rm{C}}}_{17}+{{\rm{C}}}_{19}+{{\rm{C}}}_{21}+{{\rm{C}}}_{29}+{{\rm{C}}}_{31}+{{\rm{C}}}_{33})$$

The vascular plant endmember for the TAR was determined by compiling values in modern gymnosperms and angiosperms (0.83–1.00; 95% confidence intervals; *n* = 1,734)^66^. As there is no evidence that algae synthesize long-chain *n*-alkanes^67,68^, the marine endmember for the TAR was defined as 0.

The cyclization of tetramethylated branched GDGTs is defined following ref. ^69^:

3 $$\begin{array}{l}{\rm{\#}}{\mathrm{rings}}_{\mathrm{tetra}}=(\mathrm{brGDGT}-\mathrm{Ib}+2* \mathrm{brGDGT}-\mathrm{Ic})\\ \,\,\,\,\,\,\,\,\,\,\,\,\,\,\,\,\,\,\,/(\mathrm{brGDGT}-\mathrm{Ia}+\mathrm{brGDGT}-\mathrm{Ib}+\mathrm{brGDGT}-\mathrm{Ic})\end{array}$$

Where Ia, Ib and Ic are tetramethylated brGDGTs with zero, one and two cyclopentane moieties, respectively. Values > 0.7 are considered to represent a contribution from marine in situ production^69^.

### Three-endmember mixing model

The contributions of *n* endmembers to a bulk sample can be constrained using *n* − 1 conservative tracers, plus the ‘sum-to-unity’ constraint,

4 $$\mathop{\sum }\limits_{j=1}^{n}{\rm{f}}_{\rm{j}}=1$$

where *f*_*j*_ is the fractional contribution of endmember *j* and *j* ∈ 1*… n*. This type of endmember mixing problem is classically written in matrix form as:

5 $$\left[\begin{array}{cccc}{a}_{1,1} & {a}_{1,2} & \cdots & {a}_{1,n}\\ \vdots & \vdots & \ddots & \vdots \\ {a}_{n-1,1} & {a}_{n-1,2} & \cdots & {a}_{n-1,n}\\1 & 1 & & 1\end{array}\right] \left[\begin{array}{c} {f}_{1}\\ \vdots \\ {f}_{n-1}\\ {f}_{n}\end{array}\right]=\left[\begin{array}{c} {b}_{1}\\ \vdots \\ {b}_{n-1}\\ 1\end{array}\right]$$

where *a*_*i,j*_ is the composition of conservative tracer *i* that describes endmember *j*—for example, the BIT value of the soil endmember or the TAR ratio of the plant endmember—and *b*_*i*_ is the measured value of conservative tracer *i* in a given sample. This is written more concisely as:

6 $$ \textbf{\textit{Af=b}}$$

where ***A*** is the *n* × *n* ‘design matrix’, ***f*** is the *n* × 1 vector of fractional abundances and ***b*** is the *n* × 1 vector of measured tracer values. This approach can be expanded to solve for *f*_*i*_ values of several samples simultaneously, assuming endmembers for all samples are described by the same design matrix, as

7 $$\textbf{\textit{AF=B}}$$

where

8 $$\textbf{\textit{F}}=\left[\begin{array}{cccc}\textbf{\textit{f}}_{1} & \textbf{\textit{f}}_{2} & \ldots & \textbf{\textit{f}}_{m}\end{array}\right]$$

is the *n* × *m* matrix of fractional contributions,

9 $$\textbf{\textit{B}}=\left[\begin{array}{cccc}\textbf{\textit{b}}_{1} & \textbf{\textit{b}}_{2} & \ldots & \textbf{\textit{b}}_{m}\end{array}\right]$$

is the *n* × *m* matrix of measured tracer values and *m* is the number of samples.

Assuming all *a*_*i,j*_ and *b*_*i,k*_ (where *k* ∈ 1*… m*) values are known perfectly, ***F*** can be solved by simply multiplying ***B*** by ***A***^*−*1^, the inverse of ***A***. In practice, this is instead solved using a non-negative least-squares approach, as the inversion approach is highly sensitive to noise. However, we first describe a method of overcoming the fact that some conservative tracers only inform the relative contributions of a subset of endmembers.

Some conservative tracers used here are insensitive to the fractional contributions of some endmembers. For example, the BIT index is treated as a linear mixture of *f*_soil_ and *f*_marine_ only; it is insensitive to *f*_plant_ values. This can be seen mathematically as

10 $${\mathrm{BIT}}_{\mathrm{measured}}={\mathrm{BIT}}_{\mathrm{soil}}\left(\frac{{f}_{\mathrm{soil}}}{{f}_{\mathrm{soil}}+{f}_{\mathrm{marine}}}\right)+{\mathrm{BIT}}_{\mathrm{algal}}\left(\frac{{f}_{\mathrm{marine}}}{{f}_{\mathrm{soil}}+{f}_{\mathrm{marine}}}\right)$$

We must modify our model (equations (5)–(7)) accordingly to deal with this fact. Specifically, equation (10) can be re-written as

11 $${f}_{\mathrm{soil}}{(\mathrm{BIT}}_{\mathrm{soil}}-{\mathrm{BIT}}_{\mathrm{measured}})+{f}_{\mathrm{algal}}{(\mathrm{BIT}}_{\mathrm{marine}}-{\mathrm{BIT}}_{\mathrm{measured}})=0$$

which is now a linear equation of *f*_*i*_ values. Similarly subtracting all measured results from endmember values, we re-write equation (5) as

12 $$\left[\begin{array}{cclc}({a}_{1,1}-{b}_{1}) & ({a}_{1,2}-{b}_{1}) & \cdots & ({a}_{1,n}-{b}_{1})\\ \vdots & \vdots & \ddots & \vdots \\ ({a}_{n-1,1}-{b}_{n-1}) & ({a}_{n-1,2}-{b}_{n-1}) & \cdots & ({a}_{n-1,n}-{b}_{n-1})\\ 1 & 1 & \ldots & 1\end{array}\right] \left[\begin{array}{l}{f}_{1}\\ \vdots \\ {f}_{n-1}\\ {f}_{n}\end{array}\right]=\left[\begin{array}{l}0\\ \vdots \\ 0\\ 1\end{array}\right]$$

where any (*a*_*i,j*_ − *b*_*i*_) entry is set to zero if tracer *i* is insensitive to endmember *j* (for example, BIT_plant_ − BIT_measured_ ≡ 0 because BIT_plant_ does not exist). This can similarly be written more concisely as

13 $$\textbf{\textit{Xf=z}}$$

where ***X*** = ***A*** − ***b*** (with the exception of the final row, which remains all ones for the sum-to-unity constraint) and ***z*** = [**0**1]^T^ is a vector of length *n* of all zeros except for the final entry. This can again be expanded to solve for *f*_*i*_ values of several samples simultaneously as

14 $$\left[\begin{array}{cccc}\textbf{\textit{X}}_{1} & 0 & \cdots & 0\\ 0 & \textbf{\textit{X}}_{2} & \cdots & 0\\ \vdots & \vdots & \ddots & \vdots \\ 0 & 0 & \cdots & \textbf{\textit{X}}_{m}\end{array}\right]\left[\begin{array}{c}\textbf{\textit{f}}_{1}\\ \textbf{\textit{f}}_{2}\\ \vdots \\ \textbf{\textit{f}}_{m}\end{array}\right]=\left[\begin{array}{c}\textbf{\textit{z}}\\ \textbf{\textit{z}}\\ \vdots \\\textbf{\textit{ z}}\end{array}\right]$$

Finally, we write this more concisely as:

15 $${\boldsymbol{Y\varphi =\zeta}}$$

where ***Y*** is an (*n* × *m*) × (*n* × *m*) diagonal block design matrix, ***φ*** is a matrix of size *n* × *m* containing all fractional contributions and ***ζ*** is a matrix of size *n* × *m* containing *m* entries of ***z***. Equation (15) describes our model. Rather than solving directly by inverting ***Y***—which is highly sensitive to analytical noise and endmember uncertainty, as described above—we solve equation (15) using a non-negative least-squares approach. Specifically, we find the vector ***φ*** that satisfies

16 $$\mathop{\min }\limits_{\varphi }{||}{\boldsymbol{\zeta}} -{\boldsymbol{Y\varphi}} {||}$$

subject to the constraint

17 $${\bf\varphi }_{l}\ge 0,\,\,\,\,\,\,l=1,\ldots ,n\times m$$

using the non-negative least-squares algorithm as implemented in the SciPy package for Python. Equations (16) and (17) describe the model solution that minimizes the norm of the residual error (that is, the RMSE, root mean square error) while fulfilling the constraints that each ***f***_*k*_ is non-negative and sums to unity.

To account for uncertainty in prescribed endmember compositions—and in the sum-to-unity constraint—and to estimate uncertainty on each calculated fractional contribution, we solve our model in a Monte Carlo fashion. Specifically, we draw each a_*i,j*_ value from a uniform distribution that encompasses the 90% confidence interval of all measured values for a particular tracer of a particular endmember. We additionally slightly relax the sum-to-unity constraint by adding random error, *E*, to each entry of the final row of each ***X***_*k*_ design matrix. This approach allows for, for example, small contributions by an additional, unidentified endmember. Specifically, here we allow for 5% error (that is, *E* ∈ (−0.05, 0.05)) drawn uniformly. We then solve equations (15) and (16) and repeat this process 100,000 times.

Ideally, equations (16) and (17) will yield a perfect solution (that is, RMSE = 0) for the case of *n* endmembers and *n* − 1 conservative tracers. However, if measured values are outside of the prescribed endmember range (for example, if BIT_measured_ > BIT_soil_), then RMSE will be greater than zero. We therefore only retain the 1% of solutions (that is, 1,000 iterations) that yield the lowest RMSE values for all samples. In addition to providing an estimate of uncertainty on endmember contributions, this approach also allows us to estimate which range of measured endmember compositions best describes a given sample set.

This approach was validated in the modern ocean using surface sediments from two transects along the Mississippi–Atchafalaya River coastal system. Existing multi-proxy datasets^31,70^ confirm that soil and algal OC pools are rapidly lost upon entering the Gulf of Mexico, leading to accumulation of higher plant OC further offshore and alongshore. Our model shows that the vast majority of OC in Gulf of Mexico sediments as recorded by these biomarker proxies is derived from higher plants (typically >90%; Extended Data Fig. 1). This is consistent with previous results^70^ and the observation that plant material can comprise up to ~95% of OC in riverine and coastal marine settings^37,71^. This confirms that the biomarker endmembers and mixing framework generates realistic source apportionment where independent evidence is available.

### Vascular plant, soil and marine-derived OC mass accumulation rates during the PETM

Mass accumulation rates of vascular plant, soil and marine OC (MARs; in g cm^−2^ kyr^−1^) are calculated for the latest Palaeocene and PETM using linear sedimentation rates (LSR; cm kyr^−1^), dry bulk density (*ρ*; g cm^−3^)^72^, OC measurements and the proportion of plant, soil, or marine OC (*ƒ*_*x*_) inferred via a three-endmember mixing model calculated using equation (18):

18 $${\mathrm{MAR}}={\mathrm{LSR}}\times \rho \times ({\mathrm{TOC}}/100)\times {f}_{{\mathrm{x}}}$$

where *ρ* is dry bulk density (g cm^−3^)^72^, TOC is total organic carbon concentrations (wt%) and *ƒ*_*x*_ is the proportion of plant, soil or marine OC (*ƒ*_*x*_) inferred via a three-endmember mixing model as described above. Linear sedimentation rates for the High Arctic, Tasman Sea, Tanzania, New Jersey Margin and Denmark, are obtained via refs. ^25,32,73^ (Supplementary Information). Linear sedimentation rates for Denmark are well constrained during the onset (2.2 cm kyr^−1^), body (23.8 cm kyr^−1^) and recovery (2.8 cm kyr^−1^) of the PETM^32^ but uncertain before the PETM onset. As pre-PETM sedimentation rates are unlikely to be higher than sedimentation rates during the PETM onset^28^, we assume a value of 2.2 cm kyr^−1^. TOC estimates are obtained via ref. ^25^ and ref. ^21^. Published bulk density values are only available for one site (Tasman Sea), and thus a constant *ρ* value of 2.65 g cm^−3^ was assumed across all the sites following ref. ^74^. This is justified because the densities of most common detrital minerals (quartz, feldspars and calcite) and clay minerals (including kaolinite) lie within this narrow range (2.5–2.7 g cm^−^^3^)^75,76^.

### Climate modelling experiments

We use the DeepMIP ensemble of model simulations, part of Phase 4 of the Paleoclimate Modelling Intercomparison Project (PMIP4)^77^ and integrated within CMIP6 (Coupled Model Intercomparison Project Phase 6; (ref. ^78^)). The experimental design is detailed in ref. ^79^, with an overview of large-scale climate features provided by ref. ^38^. Simulations have been performed with various models and detailed descriptions of the models and simulations are provided in ref. ^38^. All simulations were run using identical boundary conditions (for example, palaeogeography, vegetation, solar constant and so on; ref. ^35^). Each model has also been run at different atmospheric CO_2_ levels—typically ×1, ×3 and ×6 pre-industrial (PI) CO_2_, but with a subset of these, or additional atmospheric CO_2_ concentrations, chosen by some model groups^38^.

The analysed climatologies (mean annual precipitation) are based on the last 100 years of each simulation. MAP was extracted from each simulation and normalized relative to the pre-industrial ×1 CO_2_ simulation with modern geography to remove the effect of non-CO_2_ boundary conditions. We use the multi-model mean (MMM) to minimize the influence of individual model structural biases and to highlight the common forced signal. This does not eliminate uncertainty, but it provides a conservative central estimate when models share identical boundary conditions yet differ in internal physics.

The Hadley Centre model was used to quantify plant and soil OC stocks under two different CO_2_ levels (×2 and ×4 PI CO_2_). The exact model configuration (HadCM3BL-M2.1 Da) is described in detail in ref. ^41^. This model uses the MOSES2.1 (Met Office Surface Exchange Scheme) land scheme and has a dynamic vegetation and terrestrial carbon cycle scheme, TRIFFID (Top-down Representation of Interactive Foliage and Flora including Dynamics)^36,41^.

TRIFFID demonstrates skill in reproducing large-scale features of the modern land carbon budget^80^ and shows cumulative net land carbon uptake over the twenty-first century. TRIFFID can also reproduce early Eocene vegetation distributions inferred from proxy evidence^81^. The MOSES land surface scheme has general good skill when compared to modern observations in soil hydrology^82^, soil temperature^82^ and the carbon cycle^36^. We assign soil parameters in MOSES2.1 to uniform values (Supplementary Table 11) based on the United States Geological Survey (USGS) and selected soil characteristics that were the most common from non-polar continental regions (inceptisols), which show good skill at reproducing organic carbon content in these regions (mainly mid-latitudes)^83^.

The MOSES2.1 land surface scheme exchange scheme^36^ can be fully coupled to the TRIFFID dynamic vegetation model via the five plant functional types (PFTs): broadleaf trees, needleleaf trees, shrubs, C3 (temperate) grasses and C4 (tropical) grasses. The remaining four are non-vegetated surface types: urban, inland water, bare soil and ice. As the PFT structure is modern, it does not explicitly represent nutrient limitation or detailed taxonomic diversity in past climates.

Two simulations were performed: (1) a late Palaeocene simulation with atmospheric CO_2_ concentrations of 560 parts per million by volume (ppmv) and (2) a PETM simulation with atmospheric CO_2_ concentrations of 1,120 ppmv (ref. ^84^). PETM CO_2_ estimates remain uncertain, but a combination of proxy and statistical constraints^2^ support an approximate doubling that is consistent with our experimental design. Both simulations use time-dependant boundary conditions (land–sea mask, bathymetry, topography (Getech Plc) and solar luminosity (1,354.98 W m^−^^2^). Climate means are produced from the last 100 years. Each simulation is run for over 8,000 model years to ensure they are fully equilibrated in the atmosphere, land and deep ocean.

## Online content

Any methods, additional references, Nature Portfolio reporting summaries, source data, extended data, supplementary information, acknowledgements, peer review information; details of author contributions and competing interests; and statements of data and code availability are available at 10.1038/s41561-026-02012-2.

## Supplementary information

Peer Review File

Supplementary Tables Supplementary Tables 1–14.

## Source data

Source Data Fig. 1 Proportion of plant, soil and algal OC in marine sediments at each site during the PETM (Fig. 1).

Source Data Fig. 2 Mass accumulation rates of plant, algal and soil organic carbon between pre-PETM and PETM (Fig. 2).

Source Data Fig. 3 DeepMIP model ensemble-derived estimates of mean annual precipitation under different CO_2_ scenarios (Fig. 3).

Source Data Fig. 4 HadCM3L-derived estimates of plant and soil OC stocks under different CO_2_ scenarios (Fig. 4).

## Data Availability

All data used and source data for figures and tables in this study are available via NERC Environmental Data Service (EDS) National Geoscience Data Centre (10.5285/b812f06f-4920-451c-9072-bfc7115dafff). Source data are provided with this paper.

## Code availability

The code and output of the three-endmember mixing model is available via Zenodo at 10.5281/zenodo.19630720 (ref. ^30^).
