# Supplementary material for: Enhanced marine burial of terrestrial organic carbon through the Palaeocene–Eocene Thermal Maximum
Source: Nat Geosci. 2026 Jun 16;19(8):966–72. doi: 10.1038/s41561-026-02012-2 (PMC13447108; doi:10.1038/s41561-026-02012-2)
Supplement: Supplementary file 1 [file 41561_2026_2012_MOESM1_ESM.pdf]

Response to Reviews **– author response in orange – all changes refer to *Track Changes* document**

**REVIEWER #1:**

[**Editor note**: This reviewer did not supply separate comments for the author. However, they indicated to the editor that they were supportive of publication, saying the paper applied standard techniques to an interesting research question, and convincingly showed how different terrestrial organic matter contributed to marine carbon burial through the PETM.
We thank reviewer #1 for the positive feedback.

They had a few specific notes:

- Clarify that organic matter from plants is coming from terrestrial plants.
We now refer to plants as "vascular plants" throughout.

- Line 32, "can increase" is this the incorrect tense? Can implies the present.
We rephrased as followed: "Terrestrial OC burial fluxes increase ~10-to-50-fold during the PETM."

- Line 149, "events at THIS site" maybe sounds better
Amended accordingly.

**Reviewer #2 (Remarks to the Author):**

The innovative aspect of this study is the ability to provide quantitative constraints on the relative fraction of soil OC, plant OC, and marine OC, and interpret these changes in the framework of enhanced soil erosion, leading to increased soil and plant OC export during the PETM. The idea is interesting, and having the modeling component is nice…

Thank you for the positive feedback!

…but there is one key aspect that is not considered, that is sedimentary fossil carbon. Moreover, the absence of discussion on the limitations and underlying assumptions of the fully coupled climate model weakens the comprehensiveness of the study. But I think these issues could potentially be addressed by substantial revisions.

Thank you for raising these concerns. Although fossil OC is not a carbon sink, it needs to be accounted for during the PETM (see response to Q3 below). We agree that additional clarity is required for the model simulations (see response to Q6 below).

Main comments:

1) To what extent the TAR represents higher plants vs. marine OC? As the authors acknowledged, the short-chained n-alkanes are mainly derived from aquatic algae/bacteria, which includes fresh-water and ocean water sources. For long-chain alkanes to be representative of terrestrial higher plants (leaf wax and not altered by diagenesis), the carbon preference index (CPI) should be ideally above 5, but I don't think the authors provide these numbers.

Relatively high CPI values (>5) are typical of modern plants (Bush and McInerney 2013). However, lower values are reported in terrestrial surface sediments due to early diagenetic transformations. For

example, a compilation of modern peat sediments (Naafs et al., 2019) shows that CPI values range from 3 to 30 despite undergoing no thermal degradation.

During the late Palaeocene and PETM, CPI values are consistently >1 and typically between ~2–6. This indicates some degradation has taken place but is inconsistent with thermally mature OC. This supports our conclusions about the importance of a sustained higher-plant contribution to the long-chain n-alkane pool. This is now stated in the Main Text (**L119-122**).

Additionally, similar issues arise for the BIT index, branched GDGTs and crenarchael as indicators of soil OC vs. marine OC. The limitations of these organic biomarkers (e.g., uncertainty in their exact sources, modern calibrations, alterations by burial processes etc.) should be acknowledged and discussed.

Crenarchaeol is exclusively derived from ammonia-oxidising *Nitrososphaerota* and has a largely marine source (Elling et al., 2017; Pitcher et al., 2010; Sinninghe Damsté et al., 2002). In contrast, the exact source of brGDGTs can be complex and they may be produced in riverine, lacustrine, and marine settings. In the revised manuscript, we employ the weighted number of rings of the tetramethylated brGDGTs (#$rings_{tetra}$) to evaluate in situ marine brGDGT production.

This data was compiled (High Arctic, Tasman Sea) or newly generated for this manuscript (Denmark, New Jersey Margin). In each site, the average value is < 0.7, suggesting minimal in-situ marine production (see Bijl et al., 2021; Sluijs et al., 2020; Extended Data Table 13 + 14). There is no available #$rings_{tetra}$ data from Tanzania and we cannot directly exclude in-situ marine brGDGT production at this site. **However, our results from the four other study sites implies that brGDGTs are predominantly derived from the terrestrial realm (see L142-147).**

Another caveat is that brGDGTs degrade more quickly than other soil OC tracers (e.g., long-chain *n*-alkanes, lignin phenols) (see Inglis et al., 2022 and references therein), and caution is required when applying the BIT index in a system characterized by extensive organic matter degradation (e.g., large floodplains). Our low BIT values should therefore be regarded as conservative minima for soil OC input. This is clearly stated in the revised manuscript (**L146-149**).

2) Another important aspect is the fraction of the sedimentary fossil carbon, which has been shown to have played a significant role in driving the sustained CO2 emissions (i.e., Lyons et al. 2018 NG). This paper was only cited when constant bulk density values were used (not sure why they are constant, given the depositional environments are different, seems a variable bulk density would make more sense). Lyons et al. used a number of thermal maturation biomarkers, which I would imagine the authors should have the results during their biomarker analyses. If not, new analyses would be helpful to address these concerns.

If they are referring to reburial of rock OC (e.g. Hilton et al., 2011; Geology, Galy et al., 2008; Science), this could lead to an overestimate of biospheric OC burial and our mixing analysis is blind to it. Reburial of rock OC can be evaluated using published lipid biomarker thermal maturity ratios from each site (Hollingsworth et al., 2024; Stokke et al., 2020). A summary is provided below:

1) **High Arctic** – n-alkane and hopane-based thermal maturity ratios exhibit relatively stable trends throughout the sequence, <u>suggesting no change in the relative proportion of $OC_{petro}$ vs $OC_{bio}$.</u>
2) **East Tasman Plateau** - thermal maturity ratios exhibit a relatively stable trend throughout the sequence with only a subtle increase during the body of the PETM, <u>suggesting only a minimal change in the relative proportion of $OC_{petro}$ vs $OC_{bio}$.</u>

3) **New Jersey Margin** – the CPI range is similar during the pre-PETM and PETM interval. The $C_{30-31}$ αβ/(αβ + ββ) values remain relatively constant suggesting no change in the relative proportion of $OC_{petro}$ vs $OC_{bio}$. However, there is a short-lived increase in the $C_{31}$ S/(S + R) values (indicating higher thermal maturity) during the onset of the PETM before returning to pre-baseline values during the body and recovery phase.
4) **Denmark** – hopane maturity parameters were not measured at this site by Stokke et al. (2020). However, the *n*-alkanes have a constant high odd-over-even preference (OEP), with OEP >1 throughout indicating that the sequence is thermally immature suggesting no change in the relative proportion of $OC_{petro}$ vs $OC_{bio}$.
5) **Tanzania** – The CPI generally remains >3 (i.e., low thermal maturity) but there are lower CPI values and delivery of thermally mature αβ hopanes during the PETM, indicating an increased proportion of $OC_{petro}$ delivery relative to $OC_{bio}$.

The thermal maturity data indicate there is no compelling evidence for changes in **the relative proportion of $OC_{petro}$ vs $OC_{bio}$ during the PETM** at four sites (High Arctic, East Tasman Plateau, New Jersey, Denmark).This is now discussed in the revised manuscript (**see L191-204**).

Only in Tanzania is there strong evidence for a major change in OC source during the PETM, consistent with previous interpretations. However, this does not affect our interpretation because we are unable to apportion the OC pool into three different sources prior to the PETM at Tanzania and thus calculate MARs (**see L191-204**)

If the reviewer is referring to CO2 emissions from rock OC oxidation – our approach can't assess this and it's out of scope. The presence of thermally mature lipid biomarkers represents the fraction of rock OC that escaped oxidation and was ultimately buried; **it provides no direct constraint on how much rock OC that was oxidised and released as $CO_2$ during transport**. Estimating this $CO_2$ flux requires additional assumptions (e.g. erosion and export efficiencies, oxidation fractions), and current estimates therefore span a wide range (~1,000–100,000 Pg C). Although rock OC oxidation could have contributed additional $CO_2$ during the PETM, our study is not designed to resolve this question quantitatively. Instead, it represents a first step towards evaluating how the terrestrial organic carbon cycle may have modulated PETM climate.

re: dry bulk density – this was not measured for every site – as such, a fixed value of 2.65 $cm^{-3}$ was used. This simplification is justified because densities of most common detrital minerals (quartz, feldspars, and calcite) and clay minerals (including kaolinite) lie within this narrow range (2.5–2.7 $g/cm^3$). (Garzanti et al., 2008) (Salazar et al., 2017). This justification has been added to the Methods (**L551-555**).

3) Three endmember mixing models and their uncertainty. The results of this study are heavily dependent on the three end member mixing model. However, it is not clear whether this model has been successfully applied elsewhere, or this is newly developed in this work. It would be helpful to show how this is done in a more interactive way to allow others to reproduce the results.

Three end-member mixing models are widely used to apportion soil vs higher plant vs algal OC in marine sediments (Weijers et al., 2009 GCA; Gordon and Goni 2003 GCA). These studies use a wide variety of tracers to apportion soil vs higher plant vs algal OC, including elemental ratios (C/N), carbon isotopes ($^{14}C$, $^{13}C$), and biomarkers (alkanes, GDGTs, lignin phenols, alkenones). Many of these studies require only two tracers (e.g. 13C and 14C) to partition three sources (e.g., Marwick 2015 Global Biogeochemical Cycles; Mayorga 2005 Nature). However, no study – to the best of our knowledge – has used GDGTs and n-alkanes to partition three sources.

To evaluate our approach in a natural system, we applied our three end-member mixing model to biomarker data from surface sediments along two transects offshore of the Mississippi–Atchafalaya River (MAR) system. Previously generated multi-proxy datasets (Yedema et al., 2022; 2024) indicate that soil and algal OC pools are rapidly lost upon export to the Gulf of Mexico, <u>with preferential accumulation of higher-plant OC further offshore and alongshore.</u> Thus, these samples provide an ideal opportunity to assess whether our end-member definitions and mixing framework recover realistic source apportionment.

Our model indicates that Gulf of Mexico sedimentary OC recorded by our biomarker proxies is overwhelmingly derived from higher plants (typically >90%). This result agrees with existing leaf-wax constraints and with the high abundance of other plant lipids not included in the model (e.g., plant-derived sterols; Yedema et al., 2022), and is in line with prior studies showing that plant material can comprise up to ~95% of organic matter in riverine and coastal marine settings (Keil et al., 1994; Kao et al., 2014; Repasch et al., 2022). This confirms that the biomarker endmembers and mixing framework generates realistic source apportionment where independent evidence is available (**L516-527 and Extended Figure 6, shown below).**

However, there are caveats to our approach – specifically, it does not consider potential preservation biases between different compound classes. Long-chain n- alkanes exhibit strong absorption to clay minerals due to their relatively hydrophobic nature (Kusch et al., 2021), whereas GDGTs exhibit weak organo-mineral associations (Yedema et al., 2024). This is consistent with previous research in the modern-day Gulf of Mexico (Yedema et al., 2024) and the observation that n-alkanes are more resistant to degradation than other biomarkers. This is now stated in the Main Text (**L160-164**).

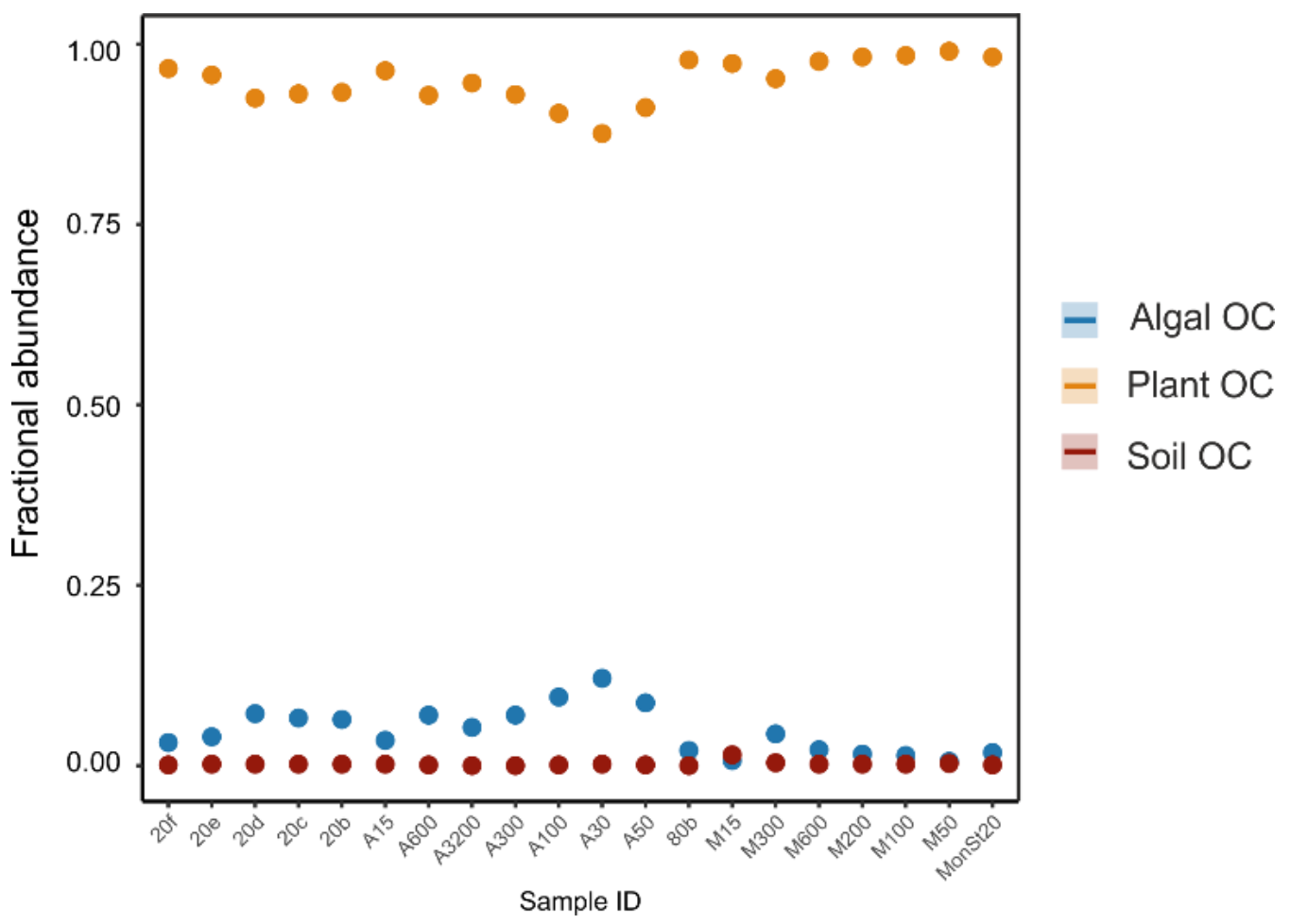


**Extended Figure 6: Fractional abundance of plant (orange), soil (red), and marine OC (blue) in core-top sediments from the Mississippi–Atchafalaya River (MAR) system**.

4) Mechanism and quantification of OC. The authors propose that changes in hydroclimate led to enhanced erosion rates, which then mobilized terrestrial OC and transported it to the river system. Figure 3 shows the complexity of this issue, as CO2 goes up, MAP goes up at three of the study sites, and goes down at two study sites (Tanzania, but Denmark MAP goes down at >1500 ppm CO2). This suggests that enhanced marine burial of terrestrial OC is not uniformly controlled by hydroclimate (i.e., the hypothesis that hydroclimate modulates terrestrial organic carbon export), and enhanced erosion has strong spatial heterogeneity. This should be discussed in more detail, and a viable mechanism other than hydroclimate should be considered.

You are correct that there are processes - beyond mean annual precipitation - that influence the marine burial of terrestrial OC. Here we can think of at least two factors that could be important:

1) Geomorphic processes

In modern settings, geomorphic processes such as landslides and overland flows can erode terrestrial organic matter from plants and soils. This is particularly important in small, steep-sided river catchments. These are enhanced by runoff and runoff intensity and thus may deliver more terrestrial OC into the marine environment (**L217-219)**

2) Frequency and intensity of individual, episodic precipitation events

In modern systems, extreme rainfall events can mobilise exceptionally large volumes of sediment and terrestrial OC (Clark et al, 2022 Nature Comms., West et al., 2010; Limn. & Ocean). During the PETM, extreme rainfall events in Tanzania intensified by up to ~70% (REF), consistent with dramatic fluctuations in biomarkers and mineralogy during the PETM (Carmichael et al., 2017 and ref. therein). This mechanism likely explains the strong terrigenous OC signal in Tanzania despite reduced MAP at higher $CO_2$ (**L229-234)**

Finally, we suggest caution in interpreting the dip in precipitation at Denmark. In the DeepMIP model ensemble, only one model simulation (CESM2.1) was successfully run at x9 PI CO2. As such, there is a much higher uncertainty on this estimate compared to those obtained from multiple model simulations (i.e. x1, x3, and x6 CO2). **We therefore revise Figure 3 to remove MAP estimates from all sites at x9 PI CO2.**

5) Thermal maturity of the studied samples. There was no mention of thermal maturity of the studied samples. I would assume these are immature, but evidence is needed to support that.

Great question – various biomarkers (e.g. alkanes, hopanes) can be used to evaluate thermal maturation.

1) Carbon preference index

Modern plants and sediments contain long-chain *n*-alkanes with an odd-over-even preference (CPI values >3 to ~30) but this is progressively lost during diagenesis (CPI ~ 1). CPI values are consistently >1 (typically ~2–6), suggesting limited thermal overprinting and only minimal diagenesis.

2) Hopane thermal maturity parameters

The ratio between different hopanes and their various stereoisomers have long been utilized as a thermal maturity proxy in the field of petroleum geochemistry (e.g. Mackenzie et al., 1980). All bacteria (except one species) synthesize hopanoids with a 17β, 21β configuration. However, this changes to a more stable βα and then αβ configuration during early diagenesis and then peak oil generation, respectively. This can be evaluated using the αβ/αβ+ββ where low values (= 0) indicate thermally immature OC, whereas high values (= 1) indicate thermally mature OC. At all sites studied here (except Tanzania), $C_{30}$ ββ/αβ+ββ ratios are <0.5 prior to, during, and after the PETM and indicate that each site has undergone minimal thermal maturation due to burial processes. This was previously reported by Hollingsworth et al. (2024) and Stokke et al. (2020) and we now state this briefly in the opening section.

3) Additional lines of evidence

There are also other lines of evidence that demonstrate shallow burial, including excellent microfossil at Tanzania (Pearson et al., 2007) and excellent terrestrial/marine palynomorph preservation in the High Arctic (Sluijs et al., 2006) and East Tasman Plateau (Contreras et al., 2014).

Taken together, these independent lines of evidence indicate that the studied intervals are thermally immature.

6) Model details are lacking. The authors cited a previously published Deep-MIP effort. Given that the community of organic geochemistry and paleoclimate modeling likely doesn't speak the same language, it would be helpful to the audience to know the details, limitations and assumptions that go into the climate models. Uncertainties associated with the estimated productivity and OC stock size should also be discussed.

We agree that additional context is needed for readers outside the paleoclimate modelling community. We have therefore provide an overview of the DeepMIP experimental design (model type, resolution, prescribed boundary conditions, and variables extracted), alongside an explicit discussion of key assumptions and limitations. We also now discuss uncertainties in model-derived productivity and terrestrial OC stock estimates and argue that these should be interpreted as first-order estimates rather than precise absolute magnitudes.

**Limitations of climate model simulations:**

---

Climate model uncertainties fall into two broad categories**:** (i) boundary-condition uncertainty, and (ii) climate-model (structural/parameter) uncertainty**.**

**Boundary condition uncertainty** includes paleogeography (e.g. topography, bathymetry, land–sea distribution), vegetation, ice sheets and greenhouse gas concentrations. Paleogeographic reconstructions rely on plate models which are relatively well constrained at 56Ma due to well preserved seafloor isochrons. However, topographic and bathymetric constraints remain uncertain. Ice sheets are not considered a major uncertainty here because the PETM represents an effectively ice-free climate state (e.g. Zachos et al., 2001). PETM $CO_2$ estimates also remain uncertain, but a combination of proxy and statistical constraints (Harper et al., 2024; PNAS) support an approximate doubling that is consistent with our experimental design.

To minimise **structural model uncertainty**, we use the DeepMIP ensemble as this applies internally consistent boundary conditions across models and reduces inter-model spread relative to earlier “ensembles of opportunity.” We also report our data (mean annual precipitation) using the multi-model mean (MMM), which minimises the influence of individual model structural biases and highlights the common forced signal. MMM are typically considered a better predictor of climate processes than single model forecasts (Tebaldi and Knutti, 2007) and are frequently used in IPCC reports. The motivation for our approach is now outlined in the Main Text (**L223-226**) and Methods (**L521-525**)

As the DeepMIP ensemble prescribes vegetation and soils it cannot directly resolve changes in terrestrial organic carbon stocks during the PETM (Figure 4). We therefore employ HadCM3BL-M2.1Da, a fully coupled atmosphere–ocean–vegetation model with dynamic vegetation (TRIFFID) and land surface scheme (MOSES2.1). The MOSES2.1 land surface exchange scheme (Cox et al., 2000) can be fully coupled to the TRIFFID dynamic vegetation model via the five plant functional types (PFTs): broadleaf trees, needleleaf trees, shrubs, C3 (temperate) grasses, and C4 (tropical) grasses. The remaining four are non-vegetated surface types: urban, inland water, bare soil, and ice. This additional level of detail has been added to the Methods (**L542-547**)

The performance and sensitivity of TRIFFID has previously been evaluated alongside four other dynamic vegetation models (Sitch et al., 2008). These all demonstrate skill in reproducing large-scale features of the modern land carbon budget (Sitch et al., 2008) and show cumulative net land carbon

uptake over the 21st century. TRIFFID has also been applied successfully to reproduce early Eocene vegetation distributions inferred from proxy evidence (Loptson et al., 2010; Climate of the Past). This is now noted in the Methods **(L583-586)**

Various papers have also quantified the performance of the MOSES land surface scheme noting that it shows in general good skill when compared to modern observations in soil hydrology (e.g. Kong et al., 2011), soil temperature (e.g. Smith et al., 2007), carbon cycle (e.g. Cox et al., 2001; Cadule et al., 2010) and perform well against modern observations of organic carbon (Collins et al., 2018). This is noted in the Methods (**L586-588**).

For deeper time simulations such as ours, uncertainties will increase because no global maps of soil characteristics for past warm climates exist. Therefore, we assign the soil parameters to uniform values (Table 1; **now Supplementary Table S11**) based on the USGS and selected soil characteristics that were the most common from non-polar continental regions (inceptisols) to use globally, which should show good skill at reproducing organic carbon content in these regions (mainly mid-latitudes) (Collins et al., 2008). This is noted on **L589**.

| **UM Name** | **Description** | **Revised Values** | **Previous Common Values** |
|---|---|---|---|
| Field329 | Wilting Point | 0.151 | 0.105 |
| Field330 | Critical Point | 0.269 | 0.17 |
| Field331 | Field Capacity | 0.32 | 0.29 |
| Field332 | Soil Saturation | 0.429 | 0.46 |
| Field1381 | Clapp—Hornberger coeff | 6.524 | 8.0 |
| Field336 | Heat Conductivity | 0.258 | 0.47 |
| Field333 | Saturated conductivity | $5.090 \times 10^{-3}$ | $3.6\times10^{-3}$ |
| Field335 | Heat Capacity | $1.248 \times 10^{6}$ | $2.22e^{-6}$ |
| Field342 | Saturated Soil Water Suction | 0.187 | 0.34 |
| Field1395 | Soil Albedo | 0.122 | 0.17 |
| Field1397 | Soil Carbon | 14.731 | 10.0 |

MOSES2.1 soil parameters used within the HadCM3B palaeoclimate model.

**Reviewer #3 (Remarks to the Author):**

This is probably one of the most interesting and well written manuscripts I have read in the last 5 years. […] I found the datasets robust, well explained, conclusions reasonable and well-supported. I think this is a major step forward in our understanding of meso-scale carbon cycle behaviors. I have no major issues with this manuscript apart from some relatively minor considerations below.

Thank you for the very kind words!

Additional components to consider:

It seems to me the nature of the CIE requires not just sequestration of any carbon, but the preferential sequestration of isotopically-light carbon in order to return to baseline d13C values in proxies. It seems likely that your study facilitates this phenomenon by sequestering plant carbon, which has a lower d13C value that other carbon sinks. Thoughts? I think it strengthens the pattern you are observing.

This is an excellent point – burial of 13C-depleted terrestrial OC may help drive atmospheric d13C values back to pre-event values quicker than 13C-enriched carbon sources (e.g. algal OC). This is consistent with Bowen and Zachos (2010) who used isotope mass balance to make a similar argument. They argued that regrowth of terrestrial OC stocks during the recovery phase of the event would have sequestered $^{13}C$-depleted carbon, contributing to the rapid CIE recovery and climate stabilization. However, biosphere regrowth alone is unlikely to be sufficient and sustained recovery on $10^4$–$10^5$ yr timescales likely requires transfer of this 13C-depleted terrestrial carbon into sedimentary reservoirs (continental and/or coastal marine sediments). The dominance of 13C-depleted higher-plant OC in coastal marine sediments therefore strengthens our interpretation that the terrestrial OC cycle played a key role in driving PETM recovery (**see L306-311**)

Minor issues:
Check references for capitalization issues particularly for journal names.

We have had a thorough check and made appropriate edits.

## Remaining reviewer comments

| Our guidance: | Your response: |
|---|---|
| REVIEWER COMMENTS: As mentioned in my email, please ensure that the code is clearly documented and commented. You're welcome to disregard the first point from the reviewer, but please be sure the address the discrepancy between the number of iterations in the code and documentation. | The code is incorrect and the number of iterations should be 100,000 – the documentation is correct. The code has been revised and revised data generated. There are no changes to the overall results. |

nature portfolio

# Peer Review File

# Enhanced marine burial of terrestrial organic carbon through the Palaeocene-Eocene Thermal Maximum

Corresponding Author: Dr Gordon Inglis

Version 0:

Reviewer comments:

Reviewer #1

(Remarks to the Author)

Reviewer #2

(Remarks to the Author)
Review for "Marine burial of terrestrial organic carbon modulates past warm climates"
This study aims to understand enhanced marine burial of terrestrial organic carbon as a viable mechanism that aids the recovery of climate following the PETM. They use a data-modeling approach, in which source-specific biomarker records from shallow marine PETM sites are generated and compiled, and the source-to-sink delivery of plant and soil OC from land to sea is inferred. A three-endmember mixing model (contrasting the previously commonly used two-end member mixing model) was used to apportion the three pools of OC (i.e., soil, plant and marine OC). Additionally, fully coupled paleoclimate models (previously published) are used to assess the mechanisms for terrestrial OC export changes across the PETM. The innovative aspect of this study is the ability to provide quantitative constraints on the relative fraction of soil OC, plant OC, and marine OC, and interpret these changes in the framework of enhanced soil erosion, leading to increased soil and plant OC export during the PETM. The idea is interesting, and having the modeling component is nice, but there is one key aspect that is not considered, that is sedimentary fossil carbon. Moreover, the absence of discussion on the limitations and underlying assumptions of the fully coupled climate model weakens the comprehensiveness of the study. But I think these issues could potentially be addressed by substantial revisions.

Main comments:
1) To what extent the TAR represents higher plants vs. marine OC. As the authors acknowledged, the short-chained n-alkanes are mainly derived from aquatic algae/bacteria, which includes fresh-water and ocean water sources. For long-chain alkanes to be representative of terrestrial higher plants (leaf wax and not altered by diagenesis), the carbon preference index (CPI) should be ideally above 5, but I don't think the authors provide these numbers. Additionally, similar issues arise for the BIT index, branched GDGTs and crenarchael as indicators of soil OC vs. marine OC. The limitations of these organic biomarkers (e.g., uncertainty in their exact sources, modern calibrations, alterations by burial processes etc.) should be acknowledged and discussed. Another important aspect is the fraction of the sedimentary fossil carbon, which has been shown to have played a significant role in driving the sustained $CO_2$ emissions (i.e., Lyons et al. 2018 NG). This paper was only cited when constant bulk density values were used (not sure why they are constant, given the depositional environments are different, seems a variable bulk density would make more sense). Lyons et al. used a number of thermal maturation biomarkers, which I would imagine the authors should have the results during their biomarker analyses. If not, new analyses would be helpful to address these concerns.
2) Three endmember mixing models and their uncertainty. The results of this study are heavily dependent on the three end member mixing model. However, it is not clear whether this model has been successfully applied elsewhere, or this is newly developed in this work. It would be helpful to show how this is done in a more interactive way to allow others to reproduce the results.
3) Mechanism and quantification of OC. The authors propose that changes in hydroclimate led to enhanced erosion rates, which then mobilized terrestrial OC and transported it to the river system. Figure 3 shows the complexity of this issue, as $CO_2$ goes up, MAP goes up at three of the study sites, and goes down at two study sites (Tanzania, but Denmark MAP goes down at >1500 ppm $CO_2$). This suggests that enhanced marine burial of terrestrial OC is not uniformly controlled by hydroclimate (i.e., the hypothesis that hydroclimate modulates terrestrial organic carbon export), and enhanced erosion has strong spatial heterogeneity. This should be discussed in more detail, and a viable mechanism other than hydroclimate should be considered.
4) Thermal maturity of the studied samples. There was no mention of thermal maturity of the studied samples. I would

assume these are immature, but evidence is needed to support that.
5) Model details are lacking. The authors cited a previously published Deep-MIP effort. Given that the community of organic geochemistry and paleoclimate modeling likely doesn't speak the same language, it would be helpful to the audience to know the details, limitations and assumptions that go into the climate models. Uncertainties associated with the estimated productivity and OC stock size should also be discussed.

Overall, this paper proposes an interesting idea of enhanced marine burial of terrestrial organic carbon, which was largely controlled by hydroclimate-induced changes in terrestrial OC export. The limitations of the biomarker proxy should be thoroughly investigated and the models should be fully described and discussed.

Reviewer #3

(Remarks to the Author)
This is probably one of the most interesting and well written manuscripts I have read in the last 5 years. Inglis et al. present an organic geochemical dataset spanning an important global warming event 56 million years ago (the PETM), and test the hypothesis that it led to changes in organic carbon burial rates. They use biomarkers to separate out terrestrial (plant and soil) organic matter and marine organic matter contributions to five stratigraphic sequences that range across low to high latitudes. They use a straightforward mixing model and estimates of mass accumulation rates to show substantial increases in terrestrial carbon burial within marine strata during the PETM. At a subset of localities, they show major increases during the recovery of the event (i.e., the main phase of carbon sequestration). The PETM is an important analog for modern anthropogenic warming and had many long-term consequences for the biosphere. The majority of previous research has focused on the "release" mechanisms (i.e., where from and how fast carbon was released to cause the PETM), but the "recovery" phase, wherein carbon is sequestered, has received much less attention. Inglis et al. address this problem and present a compelling argument that increased erosion rates on continents (via shifts in the hydroclimate) led to greater carbon burial in marginal and shallow water marine settings. This produces a negative feedback loop within the broader global carbon cycle and emphasizes a surprising geomorphic control on global climate. I found the datasets robust, well explained, conclusions reasonable and well-supported. I think this is a major step forward in our understanding of meso-scale carbon cycle behaviors. I have no major issues with this manuscript apart from some relatively minor considerations below.

Additional components to consider:
It seems to me the nature of the CIE requires not just sequestration of any carbon, but the preferential sequestration of isotopically-light carbon in order to return to baseline d13C values in proxies. It seems likely that your study facilitates this phenomenon by sequestering plant carbon, which has a lower d13C value that other carbon sinks. Thoughts? I think it strengthens the pattern you are observing.

Minor issues:
Check references for capitalization issues particularly for journal names.

Version 1:

Reviewer comments:

Reviewer #2

(Remarks to the Author)
Review on revised manuscript "Marine burial of terrestrial organic carbon modulates past warm climates"
The authors have addressed most of my previous comments. However, I have several additional suggestions that should be addressed before the manuscript can be considered for acceptance.
1) Title: The current title, "Marine burial of terrestrial organic carbon modulates past warm climates" is overly broad given that this study focuses specifically on the Paleocene-Eocene Thermal Maximum (PETM). As written, "past warm climates" implies general applicability beyond the PETM and may read as an overstatement. I recommend explicitly including PETM in the title to better match the scope of this manuscript. In addition, the verb "modulate" is potentially misleading, because it can imply a dominant or controlling role of marine burial of terrestrial organic carbon on warm climates, whereas this process is more appropriately framed as one contributing factor among multiple drivers and feedbacks. Consider replacing "modulates" with a more scoped term, such as "contributes to", to avoid implying primary control.
2) Abstract: The generalized "10- to 50- fold increase in terrestrial OC burial fluxes" ignores the 0.8-fold decrease at Tasman Sea, and the <10 fold sites in the High Arctic. The 10-50 fold increase in terrestrial OC flux only applies to New Jersey Margin and Denmark as shown in Fig. 2. The abstract should accurately reflect the overall findings of the paper.
3) I was unable to evaluate the implementation of the three end member mixing model because the manuscript does not provide the code or sufficient instructions to reproduce the calculation, including scripts, input files, and run parameters.
4) Are the model input parameters and outputs archived as open access resources? I did not see a link to access them from the manuscript. I believe Nature policy is to make all model source codes and outputs central to their research publicly available.

Overall, the manuscript contains several overstatements that should be corrected, and the authors should make the source

codes and outputs publicly available to ensure reproducibility.

Reviewer #3

(Remarks to the Author)
All my concerns were addressed by the authors.

**Open Access** This Peer Review File is licensed under a Creative Commons Attribution 4.0 International License, which permits use, sharing, adaptation, distribution and reproduction in any medium or format, as long as you give appropriate credit to the original author(s) and the source, provide a link to the Creative Commons license, and indicate if changes were made.
In cases where reviewers are anonymous, credit should be given to 'Anonymous Referee' and the source.
The images or other third party material in this Peer Review File are included in the article's Creative Commons license, unless indicated otherwise in a credit line to the material. If material is not included in the article's Creative Commons license and your intended use is not permitted by statutory regulation or exceeds the permitted use, you will need to obtain permission directly from the copyright holder.
To view a copy of this license, visit https://creativecommons.org/licenses/by/4.0/
